# Think big about developing the science

**DOI:** 10.1111/add.15568

**Published:** 2021-05-31

**Authors:** Jim McCambridge, Mary Madden

**Affiliations:** ^1^ Department of Health Sciences University of York York UK

**Keywords:** Alcohol industry, cannabis industry, cannabis legalisation, drug policy, science policy, tobacco industry

*The advent of big cannabis, tobacco and alcohol companies, and interactions between them, calls for big policy innovations that contend with major challenges to public health. Big science is also needed, in the form of major new investments in understanding the issues to inform societal responses*.

It is timely to consider what the legalized cannabis industry may now do about its potential regulation and how other addiction producers are operating in that sector. This market expansion has been long anticipated, and indeed, it has been planned for decades [1]. Adams and colleagues [2] gather and examine uncomfortable early evidence for New Zealand to demonstrate why it is appropriate to be concerned.

The cannabis industry is not a separate actor from other addiction industries and it is the big players who are of particular concern, not the small scale operators [3]. This is not new. Diageo's ongoing obligations to the thalidomide survivors, which they continue to report to the stock market, originated in a predecessor company getting in on drugs [4]. Big companies are governed by the profit imperative and legally mandated to maximize shareholder value in the United States and the United Kingdom [5]. They have extensive resources and are adept at using them to advance their own business interests. They also possess advanced understanding of complex political systems and how to navigate them [6]. Drug legalization debates are weaker if they ignore the scope for large alcohol and tobacco companies to diversify and advance their interests politically by infiltrating new markets, with detrimental consequences for public health [7].

Corporate messaging has long experimented with active consumer and policy actor persuasion; shaping preferences and leading opinion more broadly in market friendly directions [8]. Growing the cannabis market presents new opportunities for cross‐marketing to develop existing tobacco and alcohol markets and for cross‐fertilisation of political strategies. Many would agree that policy innovations are needed, extending what is already known about tobacco and alcohol [7], in developing the societal responses. Recognition of the need to regulate the nascent cannabis industry may entail more qualified endorsements of cannabis law reform measures, for example, by restricting the involvement of others in the addiction sector with proven track records of major adverse consequences for public health and society. Human rights to become intoxicated need to be safeguarded from such corporations if individual, community and population rights to health are not to be undermined.

Adams and colleagues [2] suggest that researchers have a key role to play and cite as precedents earlier research on other industries. We suggest there are further lessons available from this comparison. Alcohol and tobacco industry actors have profoundly biased what we think we know [9, 10, 11], and the implications extend to cannabis and far beyond. Tobacco industry research show what is possible in influencing policy when the research is done at the scale needed [12]. Literatures on alcohol [13] and gambling industry [14] research, and close attention to relations between sectors [3], are only now emerging and it shows in public policy. Cannabis really needs more than what we currently do as small groups of researchers “identifying, documenting and monitoring the risks of cannabis industry influence” [2]. Rather than piecemeal, reactive, data collection exercises undertaken in the margins of addiction science or public health, what is needed are major international research programmes that apply social sciences discipline‐based expertise proactively. We first need to make these topics attractive to social scientists.

Across industries it is reasonably clear what we might be interested in, and we need to build a convincing theoretical base capable of supporting scientific advances in what was once described as corporatology [15]. The potent neoliberal myths propagated by the modern transnational corporation are often quite generic in nature [8, 16]. Similarities in the narratives used by the emerging global cannabis industry and the key themes honed closely by the tobacco and alcohol industries since the 1950s [11] will be important to study carefully and, in the interests of public health, to combat [12]. Empirical research on particular industries will play key groundwork functions, but really we need to think big in developing the science. Maybe the corporate engineering of intoxication, and relatedly, other subtle influences on decision‐making, is a global challenge that deserves to be more widely recognised because the public and policy responses are themselves subverted by the threat [17, 18, 19]. Isn't it time to re‐imagine our research horizons?

## Declaration of interests

None
